# Crosstalk between alternative splicing and non-coding RNAs in hepatocellular carcinoma: from regulatory mechanism to therapeutic implications

**DOI:** 10.1016/j.ncrna.2026.07.001

**Published:** 2026-08-07

**Authors:** Delong Wang, Jingli Zhong, Zhangxiu Liao

**Affiliations:** a School of Pharmacy, Youjiang Medical University for Nationalities, Baise, Guangxi, 533000, PR China; b School of Basic Medical Sciences, Youjiang Medical University for Nationalities, Baise, Guangxi, 533000, PR China; c Guangxi Clinical Medical Research Center for Hepatobiliary Diseases, PR China; d Guangxi Higher Education Engineering Research Center for Gastrointestinal Microecology and Health, Baise, Guangxi, 533000, PR China; e Baise City Key Laboratory of Gastrointestinal Microecology and Health, Baise, Guangxi, 533000, PR China; f Key Laboratory of Basic Research and Transformation of Tumor Immunity and Infectious Diseases, Youjiang Medical University for nationalities, Baise, Guangxi, 533000, PR China

**Keywords:** Hepatocellular carcinoma, Non-coding RNAs, Alternative splicing, Post-transcriptional regulation, Malignant progression

## Abstract

Hepatocellular carcinoma (HCC) is a malignant neoplasm characterized by high incidence and mortality rates globally. Its pathogenesis and progression are intricate and multifaceted, necessitating comprehensive analysis and elucidation. Recent studies have demonstrated that non-coding RNAs (such as long non-coding RNAs, circular RNAs, and microRNAs) and alternative splicing, which are central to post-transcriptional regulation, engage in extensive regulatory interactions. These interactions form a complex regulatory network that significantly influences the malignant properties such as stemness, proliferation, invasion, metastasis, metabolic reprogramming, tumor microenvironment, and drug sensitivity in liver cancer cells. This review systematically explores the molecular mechanisms underlying the interactions between non-coding RNAs and alternative splicing events, highlighting the role of this regulatory network in modulating the expression of oncogenes and tumor suppressor genes in HCC. Additionally, this review investigates the prospective application of pivotal molecules within this regulatory network as novel diagnostic biomarkers and therapeutic targets. It also analyzes the challenges and obstacles encountered in the clinical translation process, thereby providing new insights for research on the precision diagnosis and treatment of HCC.

Primary liver cancer is a highly prevalent and lethal malignant tumor globally, which has become a major public health challenge. According to the data from “Global Cancer Statistics 2022”, it ranks 6th in terms of incidence and 3rd in terms of mortality among global cancers, with continuously increasing burden [1,2]. Hepatocellular carcinoma (HCC) accounts for approximately 90% of primary liver cancers and is the most common pathological type [3]. The clinical diagnosis and treatment of HCC face numerous difficulties: the early symptoms are insidious and non-specific, and most patients are already at the middle-to-advanced stages at the time of initial diagnosis, missing the opportunity for radical treatment; although radiotherapy, transcatheter arterial chemoembolization, targeted immunotherapy, and liver transplantation have been continuously evolving, surgical resection remains the optimal radical treatment option for early-stage patients meeting the indications [4]. Even after surgery, the 5-year survival rate of patients is only 40%– 50% [5]. The high invasiveness and dismal prognosis of HCC present substantial challenge to its clinical management. The critical bottleneck in current research and clinical practice is the paucity of reliable molecular markers for early diagnosis, coupled with incomplete characterization of pivotal molecular targets underlying its tumorigenesis and malignant progression. Therefore, it is of great and urgent significance to deeply explore the molecular regulatory mechanisms of HCC.

In recent years, post-transcriptional regulation, especially alternative splicing (AS) of precursor mRNA (pre-mRNA) and non-coding RNA (ncRNA)-mediated regulation, has become a frontier area in tumor research [6]. Increasing evidence suggests that these two pivotal regulatory systems are functionally interconnected and participate in dynamic crosstalk within a complex regulatory network [7].

AS is a process in which pre-mRNA generates multiple mRNA splicing isoforms by selectively removing introns and retaining exons [8]. Studies have confirmed that AS occurs in over 90% of multi-exon genes in humans, serving as an important source of transcriptome complexity and proteome diversity [9]. Aberrant AS is critically involved in the initiation and progression of HCC, and its associated molecules or events can serve as early diagnostic markers for HCC [10]. Daniels NJ et al. identified eight immune-related AS events by meta-analyzing peripheral blood single-cell transcriptomics, that effectively differentiate HBV-related HCC, chronic HBV infection, non-HBV liver diseases, and healthy control groups [11]. Meanwhile, modulating AS offers a novel approach for the prevention and treatment of HCC. Zhang F et al. found that inhibiting the U12 splicing factor ZCRB1 can lead to the retention of intron 11 of USP21, thereby inhibiting the malignant progression of HCC [12]. This suggests that targeting AS events may become an important strategy for the prevention and treatment of HCC.

NcRNAs mainly include long non-coding RNAs (lncRNAs), circular RNAs (circRNAs), microRNAs (miRNAs), small nucleolar RNAs (snoRNAs), transfer RNAs (tRNAs), etc. Growing evidence indicates that diverse splicing factors and splicing-regulatory RNA-binding proteins (e.g. MBNL3, DDX17, HnRNP A1/E1, SRSF1, EIF4A3, QKI, QKI 5, and AUF1) and RNA modifications collectively shape the functional diversity of ncRNA isoforms via AS. This regulation broadly influences key biological processes in HCC, such as proliferation, stemness, epithelial–mesenchymal transition (EMT), metastasis, ferroptosis, fatty acid metabolism, chemoresistance, and immune microenvironment remodeling (Table 1). In turn, numerous ncRNAs, particularly lncRNAs such as MALAT1, LINC01089, LINC01348, RAB30-DT, LINC01446, ANRIL, CLRN1-AS1, and LINC00152, regulate the expression and activity of the splicing machinery, thereby reprogramming the AS of downstream oncogenic and tumor-suppressive genes (e.g., DIAPH3, EZH2, CDCA7, VEGFA, BIM, BIN-1, TEAD-1, S6K1, ANGPT1, p73, caspase 2, SREK1, and PKM). Consequently, in addition to modulating HCC proliferation, stemness maintenance, metastasis, metabolic reprogramming, and chemoresistance, these events also affect glycolysis and tumor angiogenesis, thereby significantly driving HCC initiation and progression (Table 2). Taken together, these findings underscore the pivotal role of the “ncRNA-AS axis” in HCC pathogenesis, highlighting its dual potential as a novel molecular biomarker for early diagnosis and as a promising therapeutic target for precision intervention. This review systematically elaborates on the critical mechanisms underlying crosstalk between diverse classes of ncRNAs and AS in HCC, as well as their regulatory impacts on HCC-associated malignant biological behaviors. It also explores targeted therapeutic strategies based on these mechanisms. Through a comprehensive analysis of existing literature, it provides new perspectives and directions for the early diagnosis and precise treatment of HCC.

**Table 1:** Aberrant AS Events of ncRNAs in HCC.

Gene	Spliced isoforms	Mechanism of AS	Tumorigenic Effect	Reference
UGP2	LncRNA-SVUGP2	Unknown	Suppressing the proliferation and invasion of liver cancer cell lines in vitro and tumor growth in vivo	[13]
CASC2	LncRNA CASC2c	Unknown	Inhibiting the growth, migration, and invasion of HCC cells in vitro and promotes their apoptosis.	[14]
LncRNA-PXN-AS1	LncRNA-PXN-AS1-L	MBNL3 induces lncRNA-PXN-AS1 exon 4 inclusion	Promoting the growth and survival of HCC cells in vitro and in vivo, and inhibits apoptosis of HCC cell in vitro	[15]
	LncRNA-PXN-AS1-IR3	DDX17 induces intron 3 retention of PXN-AS1	Increasing HCC cell migration, and invasion in vitro, and enhance hepatocarcinogenesis and lung metastasis in vivo	[16]
PNUTS	LncRNA-PNUTS	HnRNP E1 inhibits the AS of lncPNUTS	Promoting HCC cancer cell metastasis and invasion	[17]
PLXNB2	LncFAL	YTHDF2 increases the splicing of lncFAL in a m6A-dependent manner	Decreasing vulnerability to ferroptosis in vitro and in vivo	[18]
CDYL	Circ-CDYL	EIF4A3 facilitates the back-splicing of circ-CDYL	Promoting HCC lung metastasis by facilitating epithelial-mesenchymal transition	[19]
RHBDD1	CircRHBDD1	EIF4A3 induces the biogenesis of circRHBDD1	Enhancing the proliferative capabilities of HCC, augmenting aerobic glycolysis and restricting anti-PD-1 therapy in vitro and in vivo	[20]
CSNK1G1	Hsa_circ_10155	EIF4A3 increases hsa_circ_101555 expression by combining with the flanking sequences	Promoting the proliferation, migration, and invasion of HCC cells in vitro and in vivo	[21]
Unknown	Circ-0008150 circ-0007821	QKI causes the formation of circ-0008150 and circ-0007821	Increasing cell invasion ability by promote EMT progression	[22]
EPB41	CircEPB41(2	Glucose deprivation induces cirEPB41(2) expression in an HNRNPA1-dependent manner	Enhancing the proliferation of HCC cells and in vivo growth of tumor xenografts by promoting lipogenesis	[23]
PRKAA1	CircPRKAA1	Low AMPK activation status induces circPRKAA1 expression in an SRSF1-dependent manner	Increasing fatty acid synthesis to promote HCC cell growth	[24]
ZKSCAN1	CircZKSCAN1	Abnormal downregulation of QKI 5 in HCC tissues contributes to the reduced expression of circZKSCAN1	Suppressing malignant behavior of HCC by modulating cell stemness	[25]
Unknown	Hsa_circ_0007874 Hsa_circ_0004913	NUDT21 increases the level of circRNAs with UGUA motifs in HCC cells	Inhibiting the proliferation of HCC cells proliferation	[26]
LncRNA MALAT1	Circ-MALAT1	RNA binding proteins AUF1 increases the biogenesis of circ-MALAT1	Promoting the self-renewal of HCC cancer stem cells	[27]
UBXN7 SLC22A3	Hsa_circ_0001380 Hsa_circ_0078607	CPSF3 promotes the shift of pre-mRNA containing the PAS element from circRNA to linear mRNA in HCC cells	Promoting HCC cell proliferation and migration	[28]
ARL3	Circ-ARL3	HBx protein favors circARL3 splicing and biogenesis attributed to m^6^A modification	Increasing the proliferation and invasion of HBV^+^ HCC cells.	[29]
PSD3	CircPSD3	CircPSD3 is up-regulated in HCV-infected liver cells	Displaying pro-viral effects	[30]
C13orf25	miR-17、miR-20、miR-92a	SLU7 binds to C13orf25 transcripts in vivo and increasing splicing of pri-miR-17-92	Contributing to HCC survival by reducing ROS and autophagy-related apoptosis	[31]
tRNA-Glu/TTC	HCETSR	Spliceosome and dicer1 promote the splicing of tRNA-Glu/TTC in HCC	Inhibiting the Malignancy of HCC in vitro and in vivo	[32]

**Table 2:** Regulatory Roles of ncRNAs in AS in HCC.

NcRNAs	Mechanism of AS	Splicing targets	Tumorigenic Effect	Reference
LncRNA MALAT1	MALAT1 stabilizes PSF interaction with several hnRNPs and PTBP1, thereby forming a functional module that affects a network of AS events.	Unknown	Unknown	[33]
LncRNA LINC01089	LINC01089 interacted with hnRNPM and led to hnRNPM-mediated skipping of DIAPH3 exon 3	DIAPH3	Promoting EMT, migration, invasion and metastasis of HCC cells in vivo and in vitro	[34]
LncRNA LINC01532	LINC01532 bound to hnRNPK and promoted CDK2-mediated phosphorylation of hnRNPK, which facilitated G6PD pre-mRNA splicing	G6PD	Blunting lenvatinib-induced cell death, leading to drug resistance	[35]
LncRNA LINC01348	LINC01348 complexed with SF3B3 acted as a modulator of EZH2 pre-mRNA AS	EZH2	Inducing marked inhibition of cell growth, and metastasis in vitro and in vivo	[36]
LncRNA RAB30-DT	RAB30-DT directly binds and stabilizes the splicing kinase SRPK1, facilitating its nuclear localization	CDCA7	Promoting HCC cell proliferation, migration, invasion, colony and sphere formation in vitro, and tumor growth in vivo	[37]
LncRNA LINC01446	LINC01446 bound to SRPK2 and activated its downstream target, SRSF1	VEGFA	Promoting HCC growth, angiogenesis and chemoresistance in vitro and in vivo	[38]
LncRNA MALAT1	Induction of SRSF1 by MALAT1 modulates SRSF1 splicing targets	BIM, BIN-1, TEAD-1, and S6K1	Inducing transformation and tumorigenesis of HCC cells in vitro and in vivo	[39]
LncRNA ANRIL	ANRIL acts as a sponger of miRNA‐199a‐5p, resulting in an elevated level of its target protein SRSF1	Unknown	Inducing HCC cell proliferation, mobility, tumorigenesis, and metastasis in vitro and in vivo	[40]
LncRNA CLRN1-AS1	CLRN1-AS1 binds to miR-320c, which targets the expression of SRSF7	Unknown	Inhibiting the proliferation and migration of HCC cells	[41]
LncRNA LINC00152	Interaction of Trx and LINC00152 promots EMT via regulating alternative splicing of ANGPT1	ANGPT1	Promoting migration and invasion of HCC cells in vitro and increase the rate of lung metastasis of HCC cells in vivo	[42]
EVA1A-AS	Eva1A-AS inhibits the second intron splicing of EVA1A in a U2 dependent manner	EVA1A	Promoting HCC cell proliferation	[43]
Circ_0029343	Circ_0029343 could sponge miR‐486‐5p to up‐regulate SRSF3	p73	Promoting the proliferation, migration, and invasion and inhibiting apoptosis of HCC cells in vitro and the growth and metastasis of HCC cells in vivo	[44]
MiR-193a-3p	MiR-193a-3p represses the expression of SRSF2 and downregulates the proapoptotic splicing form CASP2L	Caspase 2	Dictating the 5-FU resistance of HCC cells	[45]
MiR-30c/e−5p	MiR-30c-5p and miR-30e-5p inhibit the expression of SRSF10 and the splicing of SREK1	SREK1	Inhibiting HCC CSC	[46]
MiR-374b	MiR-374b reduces the expression of hnRNPA1 by binding to its 3′ untranslated region	PKM2	Re-sensitizing sorafenib-resistant HCC cells and mouse xenografts to sorafenib treatment	[47]
MiR-133b	MiRNA-133b negatively regulated the expression of SF3B4	Unknown	A tumor suppressor in HCC	[48]
MiR-320c	MiR-320c inhibits the expression of SRSF7	Unknown	Promoting the proliferation and migration of HCC cells	[41]
MiR-1183	MiR-1183 function as a tumor suppressor by specifically binding to SRSF1.	Unknown	Weakening proliferation ability and migration ability in vitro and inhibiting subcutaneous tumor formation in vivo	[49]
MiR-486-5p	MiR-486-5p interacts with SRSF3	Unknown	Exhibiting anti-cancer properties both in vitro and in vivo	[50]

## 1 The process of AS and its regulatory factors

**Fig. 1:**
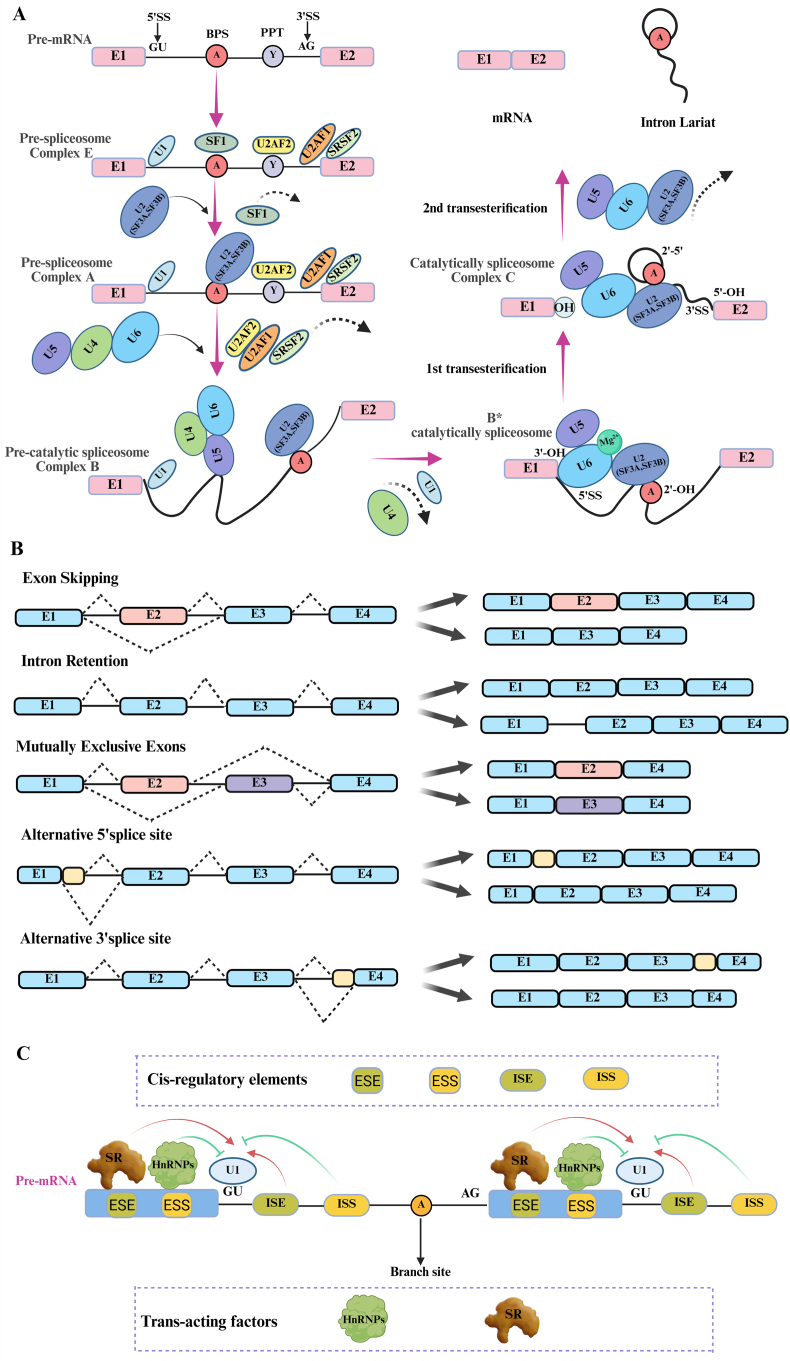
**Process, basic patterns and regulatory mechanism of alternative splicing.** (A) Spliceosome-mediated pre-mRNA splicing process: First, U1 snRNP recognizes the 5′ splice site, U2AF proteins (U2AF1/U2AF2) bind to the 3′ splice site and polypyrimidine tract (PPT) respectively, SR protein (SRSF2) assists in stabilizing protein-RNA interactions, and SF1 binds to the branch point sequence (BPS), collectively promoting the assembly of spliceosomal complex E. Subsequently, SF1 is released, allowing U2 snRNP to bind to BPS and be stabilized by SF3A/SF3B, forming complex A. Next, the U4/U6-U5 tri-snRNP complex is recruited, and U5 snRNP recognizes exon boundaries to form complex B; thereafter, U1 and U4 snRNPs dissociate, forming the catalytically active spliceosome. Finally, two transesterification reactions occur, ultimately generating mature mRNA and the intron lariat structure. (B) Basic patterns of alternative splicing: There are five common types of RNA splicing, namely exon skipping, intron retention, mutually exclusive exons, alternative 5′ splice site selection, and alternative 3′ splice site selection. (C) Molecular mechanism of alternative splicing regulation and splice site recognition: after trans-acting factors such as hnRNPs and SR proteins recognize the corresponding cis-elements, they cooperatively assemble into a functional spliceosome to achieve precise recognition and catalysis of splice sites. This regulatory network determines the inclusion or skipping of exons and introns through the competitive balance between splicing enhancers and silencers, which is the core mechanism for generating alternative splicing diversity.

As a large multi-subunit ribonucleoprotein (RNP) complex, the spliceosome constitutes the central effector complex governing. It comprises five small nuclear ribonucleoproteins (snRNPs): U1, U2, U4, U5, and U6. Upon recognizing the 5′ splice site (harboring the conserved GU dinucleotide), 3′ splice site (bearing the conserved AG dinucleotide), branch-point sequence containing a critical adenosine residue, and polypyrimidine tract within pre-mRNA, the spliceosomal components sequentially assemble into a functional catalytic complex. Subsequently, two consecutive transesterification reactions give rise to a diverse array of AS events, encompassing exon skipping (the most predominant type), intron retention, mutually exclusive splicing, and alternative 5′ or 3′ sites selection. The specific processes and types are illustrated in Fig. 1A and B [[51], [52], [53]].

The fundamental regulation of AS hinges on the intricate interplay between cis-acting elements and trans-acting splicing factors. Cis-acting elements, such as intronic splicing enhancers (ISE), intronic splicing silencers (ISS), exonic splicing enhancers (ESE), and exonic splicing silencers (ESS), are specific sequences located on the pre-mRNA [54]. The principal splicing factors comprise the serine/arginine-rich (SR) protein family, which facilitates splicing, and the heterogeneous nuclear ribonucleoprotein (hnRNP) protein family, which mainly suppresses splicing. The SR protein family includes members such as SRSF1, SRSF3, and SRSF6, all of which possess an RNA recognition motif (RRM) and an RS domain. The RRM specifically binds to conserved sequences within ESEs (located in exons) or ISEs (located in introns). Binding to ESEs enables SR proteins to recruit spliceosomal factors such as U1 snRNP and U2AF through their RS domains, stabilizing spliceosome assembly at nearby splice sites and reinforcing exon inclusion. Moreover, a subset of SR proteins can also enhance splicing upon binding to ISE sequences [55]. Representative members of the hnRNP family include hnRNP A1, hnRNP H, and hnRNP U, among others, which generally lack an RS domain. Upon binding to ESS, these proteins impede splicing through steric hindrance or competition with SR proteins, leading to exon skipping. Similarly, binding to ISS obstructs the early assembly of the spliceosome, further promoting exon skipping or intron retention [56,57]. Of note, a small subset of hnRNP proteins have also been shown to recognize to ESEs under specific contexts [58]. These cis-elements and splicing factors collectively establish a well-coordinated regulatory network. The relative concentration and binding affinity of SR proteins and hnRNP proteins play a pivotal role in sustaining tissue- and spatiotemporal-specific AS patterns. AS regulation is detailed in Fig. 1C.

## 2 Aberrant AS events in HCC

Normal hepatocytes undergo constitutive AS events, which are essential for maintaining liver homeostasis and normal physiological functions [59]. In contrast, aberrant AS contributes to the progression of liver diseases, particularly the malignant development of HCC [60,61]. A growing body of evidence indicates that the AS landscape of HCC differs significantly from that of normal hepatocytes [[62], [63], [64]].

Tremblay et al. demonstrated that the AS profiles of HBV-related, HCV-related, and non-viral HCC exhibit distinct splicing aberration patterns. Specifically, HBV-associated HCC presents aberrant splicing in 761 unique transcripts, whereas HCV-associated HCC and non-viral HCC display 68 and 299 such transcripts, respectively. This finding provides a promising foundation for the development of etiologically tailored diagnostic biomarkers and therapeutic targets based on AS events [62].

Li et al. analyzed 631 RNA-seq libraries and demonstrated that differential AS events in HCC are strongly correlated with multiple core cancer hallmarks, including the cell cycle, precursor metabolite and energy metabolism, DNA damage repair, the p53 signaling pathway, EMT, cell proliferation and the TGF-β signaling pathway, with the most striking enrichment in metabolism-related pathways. Subsequent univariate and multivariate Cox regression analyses identified 243 splicing events that were independently correlated with patient overall survival, of which over two-thirds were identified as unfavorable prognostic factors [63]. These findings underscore the potential of tumor splicing patterns as molecular readout for more accurate risk stratification and survival prediction in clinical practice.

By analyzing 370 HCC samples retrieved from the TCGA database, Yu et al. identified 467 differentially expressed alternative splicing events (DEAS) that significantly correlate with immune and stromal scores. These DEAS remodel the HCC immune microenvironment and are closely associated with poor patient prognosis, suggesting that HCC cells depend on these splicing events to escape anti-tumor immune surveillance [64].

Further single-gene targeted studies have demonstrated that abnormal AS of key genes such as CD44 [65], VEGFA [38] and PKM [47] directly mediates HCC invasion, metastasis, glycolytic reprogramming, chemoresistance and tumor angiogenesis, thereby corroborating the central regulatory role of splicing dysregulation in HCC.

Cumulatively, the aforementioned evidence illustrates that these differential AS events in HCC are closely associated with metabolic reprogramming, immune microenvironment remodeling and the metastatic capacity of HCC, and hold promise as prognostic biomarkers.

## 3 The interplay between ncRNAs and AS in HCC

### 3.1 Crosstalk between AS and lncRNAs

LncRNAs are a class of endogenous non-coding RNAs longer than 200 nt. These molecules exert pro-tumor or anti-tumor functions in HCC by modulating gene transcription, AS, or serving as molecular sponges to sequester miRNAs. Their regulatory roles impact various cellular processes including proliferation, apoptosis, migration, metabolic reprogramming, the tumor microenvironment, and drug sensitivity [66].

#### 3.1.1 Aberrant AS drives distinct lncRNA isoforms with functional impact on HCC progression

Yao ZC et al. uncovered that the AS events of lncRNAs in serum exosomes of HCC patients are significantly less abundant than in healthy controls. Strikingly, two specific AS-generated isoforms—lnc-FAM72D-3 and lnc-GPR89B-15—were substantially enriched in HCC samples, while lnc-ZEB2-19 and lnc-EPC1-4 were significantly reduced [67]. Additionally, the lncRNA-SVUGP2 and lncRNA CASC2c, derived from AS of the UGP2 and CASC2 genes, respectively, are expressed at low levels in HCC and have been shown to exert inhibitory effects on the proliferation and invasion of cancer cells [13,14]. These observations highlight the existence of disease-specific AS signatures of lncRNAs in HCC, implying that AS-driven lncRNA diversification may orchestrate tumor onset and progression. Nonetheless, the upstream regulatory networks and downstream functional consequences of AS alterations in HCC remain largely elusive.

Studies have demonstrated that AS of pre-lncRNA generates multiple structural variants, which can reshape lncRNA functionality by altering their subcellular localization [68], stability [69], and their molecular interaction capacity [15]. For example, AS can drive the redistribution of lncRNAs—as observed for lncRNA OIP5-AS1 and its splicing isoform ENST00000501665.2 between the cytoplasm and nucleus [68]—or modulate their stability, as exemplified by lncRNA MIR222HG, whose turnover is regulated via competition with microRNA processing machinery [69]. Despite these findings, whether these alternative regulatory paradigms—specifically, AS-mediated alterations in lncRNA localization and stability—are functional in HCC remains to be elucidated.

**Fig. 2:**
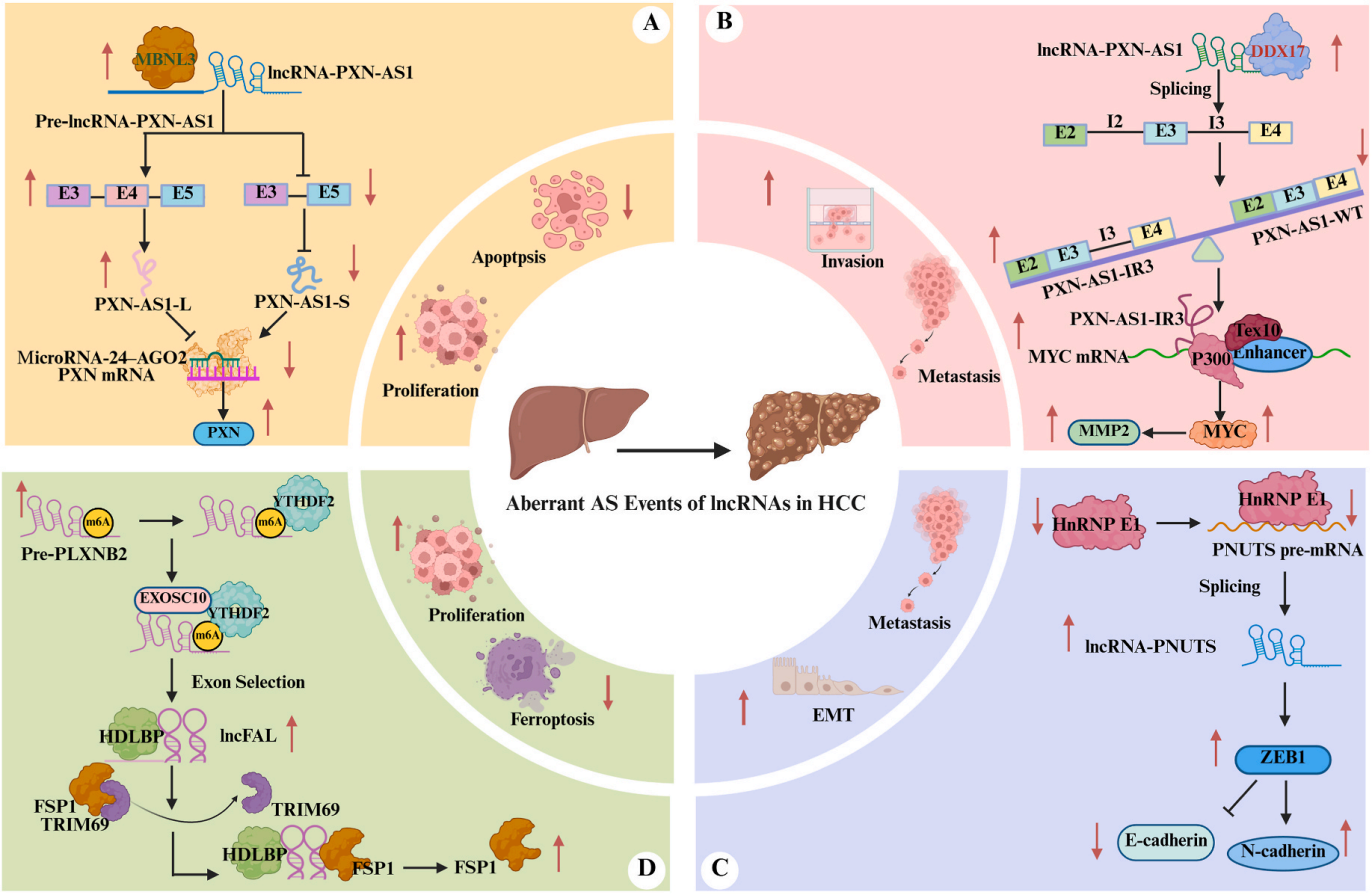
**Regulatory mechanism of abnormal lncRNA alternative splicing events in Hepatocellular Carcinoma (HCC).** The splicing factors MBNL3 (A), DDX17 (B), and hnRNP E1 (C) regulate the alternative splicing of lncRNA-PXN-AS1 and lncRNA-PNUTs, respectively, promoting the generation of oncogenic lncRNA isoforms. This drives hepatocellular carcinoma cell proliferation, epithelial-mesenchymal transition (EMT), invasion, and metastasis, while inhibiting apoptosis. Meanwhile, YTHDF2 recognizes m6A modification sites on pre-PLXNB2, facilitating its directional splicing into lncFAL. The elevated lncFAL forms a stable complex with HDLBP and FSP1 proteins, which prevents TRIM69 from binding to FSP1 and reduces TRIM69-mediated degradation of FSP1, leading to upregulation of FSP1 expression. This ultimately promotes hepatocellular carcinoma proliferation and suppresses ferroptosis (D).

Cumulative evidence indicates that AS-generated lncRNA isoforms in HCC predominantly exert their functions by modulating molecular interactions to influence tumor progression. A prime example is lncRNA-PXN-AS1, which gives rise to two functionally distinct splicing isoforms through different regulatory mechanisms [15,16]. On one hand, MBNL3-mediated exon 4 inclusion generates PXN-AS1-L, which binds to the 3′-UTR of PXN mRNA, shielding it from miR-24-AGO2-dependent degradation—a process that promotes HCC proliferation and suppresses apoptosis (Fig. 2A) [15]. On the other hand, DDX17-facilitated intron 3 retention produces PXN-AS1-IR3, which recruits TEX10 and p300 to the MYC enhancer, activates MYC transcription, and ultimately drives HCC metastasis (Fig. 2B) [16]. Similarly, Grelet S demonstrated that the splicing factor hnRNPE1 modulates the AS of the 3′ splicing site (3′SS) of PNUTS pre-mRNA; its downregulation increases lncPNUTS isoforms, upregulates ZEB1 to activate EMT, and promotes HCC metastasis (Fig. 2C) [17]. Beyond protein-mediated regulation, epigenetic mechanisms also contribute to AS of lncRNA. The m6A-binding protein YTHDF2 recognizes the "GGAC" motif—a specific m6A modification site—on pre-lncFAL, thereby facilitating the directional splicing of PLXNB2 pre-mRNA into lncFAL, which binds to the ferroptosis-related protein FSP1, enhances its stability, and suppresses ferroptosis in HCC cells (Fig. 2D) [18]. This finding highlights the involvement of epitranscriptomic mechanisms in the AS regulation of lncRNAs.

In summary, these findings suggest that the dysregulated expression of splicing factors such as MBNL3, DDX17, and hnRNPE1 in HCC can influence HCC development by regulating the AS of lncRNAs (Fig. 2). However, further investigations are warranted to determine whether additional splicing factors and spliceosomal complex assembly are involved in AS regulation of lncRNA and its subsequent impact on HCC.

#### 3.1.2 LncRNA-Orchestrated dysregulation of AS: a pivotal driver of HCC progression

**Fig. 3:**
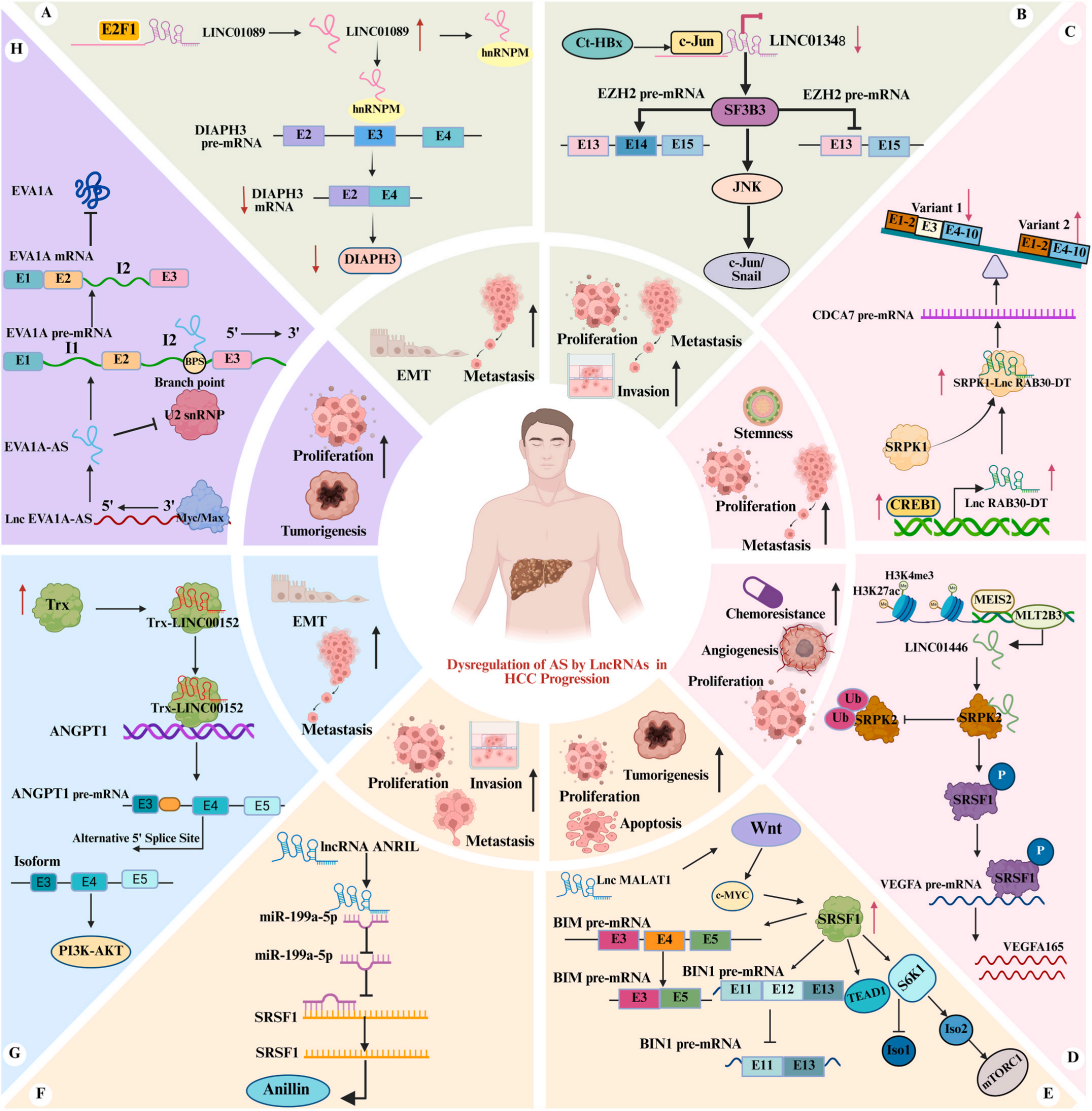
**Multiple mechanisms of lncRNA-regulated alternative splicing in hepatocellular carcinoma progression.** (A, B) LINC01089 and LINC01348 act as molecular scaffolds to bind splicing factors, regulating the alternative splicing of DIAPH3 and EZH2, respectively. This further promotes epithelial-mesenchymal transition (EMT) and metastasis, and inhibits proliferation, migration and invasion of hepatocellular carcinoma, respectively. (C, D)LncRNA RAB30-DT and LINC01446 bind to splicing kinases SRPK1 and SRPK2, respectively, promoting the production of oncogenic isoforms of CDCA7 and VEGFA, thereby facilitating hepatocellular carcinoma cell proliferation, maintaining stemness, promoting angiogenesis and migration. (E, F) LncRNA MALAT1 and ANRIL indirectly regulate alternative splicing of multiple targets through transcriptional regulation or ceRNA network, respectively, accelerating hepatocellular carcinoma proliferation and tumor progression. (G) LINC00152 binds to RNA-binding protein Trx, regulating the alternative 5′ splice site selection of ANGPT1 and promoting EMT and migration of hepatocellular carcinoma cells. (H) LncRNA EVA1A-AS forms complementary pairing with intron 2 of EVA1A pre-mRNA, masking the branch point and preventing U2 snRNP recognition, leading to intron 2 retention and ultimately promoting hepatocellular carcinoma proliferation and survival.

Wang JU et al. uncovered 1375 significant AS events in HCC by interrogating RNA-seq data from 369 HCC patients and 160 normal liver tissues, and pinpointed 31 lncRNAs associated with AS from the multi-omics network using the RWMG model, suggesting a previously underappreciated role of lncRNAs in HCC via AS regulation [70]. Emerging evidence has demonstrated that lncRNAs orchestrate AS and fuel HCC progression through diverse mechanisms. These include direct binding to splicing factors (Fig. 3A–B) [34,36] or kinases (Fig. 3C–D) [37,38] to fine-tune their activity, indirect modulation of splicing factor expression via transcription-dependent or competing endogenous RNA (ceRNA) networks (Fig. 3E–F) [39,40], assembly into functional complexes with RNA-binding proteins (RBPs) (Fig. 3G) [42], and direct regulation of spliceosome components (Fig. 3H) [43].

LncRNA can serve as molecular scaffolds to interact with specific splicing factors, modulating the formation and activity of the splicing complex. This process regulates AS of pre-mRNA, influencing HCC development. Miao H et al. reported that lncRNA MALAT1 does not alter the expression levels of splicing factors PTBP1 and PSF. Instead, it stabilizes the interaction between PTBP1 and PSF and guides this complex to target and bind to the pre-mRNA of target genes, collectively regulating the AS events of 258 exons in HCC [33]. However, this study did not identify the specific regulated genes and the direct impact of AS events on HCC malignancy, necessitating further investigation into the underlying molecular mechanisms. In another instance, LncRNA LINC01089 binds to splicing factor hnRNPM, facilitating exon 3 skipping in the DIAPH3 gene, which harbors m^6^A modification sites. This action reduces DIAPH3 mRNA stability and protein expression, thus promoting EMT and metastasis in HCC cells through activating the ERK/Elk1/Snail pathways (Fig. 3A) [34]. In a similar manner, LINC01532 functions as a molecular scaffold by concurrently interacting with hnRNPK and CDK2, which promotes CDK2-dependent phosphorylation of hnRNPK and ultimately abrogates the inhibitory effect of hnRNPK on G6PD pre-mRNA splicing, consequently suppressing both sensitivity to lenvatinib, and cuproptosis in HCC [35]. In parallel, lncRNA LINC01348 binds to the splicing factor SF3B3 in HCC, thereby disrupting the splicing of EZH2 pre-mRNA and ultimately suppressing the production of the EZH2-14+ isoform. Consequently, this inhibition suppresses the JNK/c-Jun/Snail pathway, thereby inhibiting EMT in HCC and metastasis (Fig. 3B) [36].

In addition to interacting with splicing factors, lncRNAs also associate with splicing kinases and govern their stability, subcellular localization, thus indirectly modulating splicing complexes and AS. Prominently, LncRNA RAB30-DT directly binds to and stabilizes the splicing kinase SRPK1, promoting its nuclear localization. Consequently, it regulates the AS of the cell cycle regulator CDCA7 by facilitating exon 3 skipping, which generates variant 2. This process significantly increases the tumor sphere-forming capacity of liver cancer cells in vitro and markedly upregulates stem cell marker genes such as CD133 and SOX2, both in vitro and in vivo, and modulates cellular sensitivity to multiple drugs, including dasatinib and selumetinib. This effect can be reversed by silencing the splicing kinase SRPK1 (Fig. 3C) [37]. Similarly, lncRNA LINC01446 binds to splicing kinase SRPK2, preventing its ubiquitination and degradation, while enhancing SRPK2-mediated phosphorylation and nuclear translocation of the splicing factor SRSF1. This interaction promotes the splicing of VEGFA pre-mRNA to yield the pro-vascular subtype VEGFA_165_ and reshapes the vascular microenvironment, inducing sorafenib resistance (Fig. 3D) [38].

LncRNAs can indirectly influence the splicing landscape of HCC by regulating the transcriptional levels of splicing factors. Specifically, lncRNA MALAT1 activates the Wnt signaling pathway, which induces the expression of c-MYC and cyclin D1, subsequently promoting the transcription of the splicing factor SRSF1. This cascade ultimately regulates the AS of downstream genes such as BIM, BIN1, TEAD1, and S6K1, leading to the generation of pro-cancer isoforms (Fig. 3E) [39]. Moreover, lncRNAs can counteract miRNA-mediated inhibition of specific splicing factors through ceRNA interactions, acting as miRNA sponges. For example, lncRNA ANRIL mitigates the inhibition of SRSF1 by sponging miR-199a-5p, thereby influencing the splicing of the Anillin gene via SRSF1 and promoting HCC progression (Fig. 3F) [40]. However, the specific splicing isoforms of Anillin regulated by SRSF1 require further investigation. Conversely, lncRNA CLRN1-AS1 stabilizes the expression of splicing factor SRSF7 by sponging miR-320c, exerting an anti-cancer effect. Nonetheless, the specific AS events affecting its target genes remain undefined [41].

Beyond their regulation of AS through canonical splicing factors (e.g., hnRNPM, SF3B3, SRSF1, and SRSF7), lncRNAs in HCC further reshape the AS landscape of target genes by engaging a broader spectrum of RBPs distinct from the conventional splicing machinery. For instance, lncRNA LINC00152 binds to the RNA-binding protein Trx, thereby regulating the AS of the angiopoietin 1 (ANGPT1) gene. This splicing switch activates the PI3K-Akt signaling pathway and induces EMT, ultimately promoting the invasion and lung metastasis in HCC (Fig. 3G) [42]. Even so, this specific regulatory layer, in which lncRNAs cooperate with other RBPs beyond the classical splicing factor repertoire, remains relatively underexplored and warrants further systematic investigation.

LncRNAs can also modulate the AS process in HCC by influencing spliceosome component activities. In particular, the transcript of EVA1A-AS can form complementary RNA duplexes with the second intron (I2) of the host gene EVA1A pre-mRNA. This interaction interferes with the recognition of splicing sites by U2 snRNP, and impeding the accurate splicing of I2. Consequently, this disruption hinders the production and nuclear export of its mature mRNA. As a result, the reduced splicing leads to diminished expression of the crucial tumor suppressor protein EVA1A, which in turn promotes the survival and proliferation of HCC cells (Fig. 3H) [43].

Together, existing evidence highlights that lncRNAs reshape the splicing profile of HCC cells in a highly specific manner through dynamic association with and fine-tuning of diverse splicing regulators, such as splicing factors, splicing kinases, RBPs, and spliceosome components. This process leads to the modulation of key oncogenes or tumor suppressor gene isoforms, offering novel insights into molecular mechanisms, as well as promising diagnostic and therapeutic avenues for HCC.

### 3.2 Crosstalk between AS and circRNAs

#### 3.2.1 CircRNAs generated by back-splicing modulate the progression of HCC

**Fig. 4:**
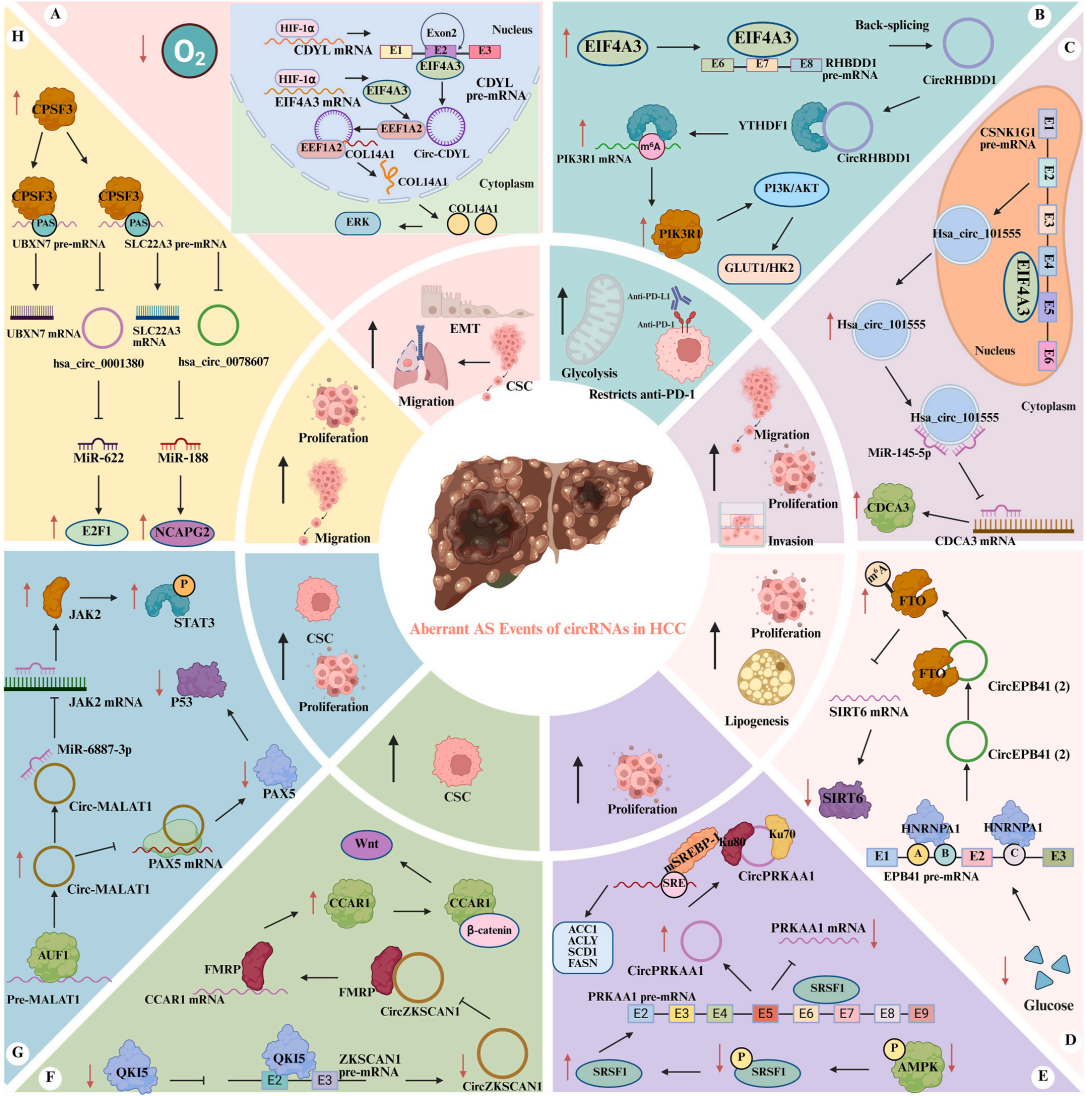
**Regulatory mechanisms of abnormal circRNA alternative splicing events in hepatocellular carcinoma (HCC).** (A) Hypoxia-induced HIF-1α activates CDYL and EIF4A3 transcription; EIF4A3 mediates CDYL pre-mRNA back-splicing to form Circ-CDYL, which upregulates COL14A1, activates ERK signaling, and promotes HCC epithelial-mesenchymal transition (EMT) and liver circulating cancer stem cell (CCSC) lung metastasis. (B) Upregulated EIF4A3 induces RHBDD1 pre-mRNA back-splicing to generate CircRHBDD1, which facilitates YTHDF1 recognition of PIK3R1 mRNA m6A modification, upregulates PIK3R1, activates PI3K/AKT pathway, and thus promotes HCC cell glycolysis and impairs anti-PD-1 immunotherapy response. (C) EIF4A3 regulates CSNK1G1 pre-mRNA back-splicing to produce Hsa_circ_101555, which upregulates CDCA3 via ceRNA network and promotes HCC proliferation, migration and invasion. (D) Reduced glucose concentration promotes HNRNPA1 binding to EPB41 pre-mRNA, inducing its back-splicing into CircEPB41 (2); CircEPB41 (2) cooperates with FTO to negatively regulate SIRT6 mRNA stability, and promotes HCC proliferation and lipid production. (E) Decreased AMPK phosphorylation increases SRSF1 dephosphorylation; dephosphorylated SRSF1 enhances PRKAA1 pre-mRNA back-splicing to produce more CircPRKAA1, which binds Ku70/Ku80/mSREBP-1 complex, upregulates lipid-related genes, and promotes HCC proliferation. (F) Decreased QKI5 expression inhibits its binding to ZKSCAN1 pre-mRNA, reducing CircZKSCAN1 production; reduced CircZKSCAN1 promotes FMRP-CCAR1 mRNA binding, upregulates CCAR1, activates Wnt pathway, and increases CSC number. (G) AUF1 promotes pre-MALAT1 back-splicing to form Circ-MALAT1, which sponges miR-6887-3p, upregulates JAK2/STAT3, downregulates PAX5 and p53, and promotes HCC cell proliferation and CSC maintenance. (H) Increased CPSF3 promotes UBXN7 and SLC22A3 pre-mRNA binding, inhibiting hsa_circ_0001380 and hsa_circ_0078607 production; their downregulation upregulates E2F1 and NCAPG2, respectively, and drives HCC proliferation and migration.

CircRNAs are a type of RNAs characterized by covalently closed circular structures, and while most are non-coding, a subset has been shown to indeed possesses protein-coding potential via cap-independent translation mechanisms [71,72]. Their generation primarily occurs through back-splicing, a unique form of AS that is meticulously regulated by cis-acting elements, such as flanking intron complementary sequences, and trans-acting factors, including RBPs and splicing factors [73]. In HCC, multiple RBPs and splicing factors modulate circRNA expression by regulating back splicing, influencing tumor progression (Fig. 4). Research has demonstrated that specific RBPs, such as NONO, HNRNPK, and ESRP2, exhibit differential expression in HCC, underscoring their role in HCC development [74]. The splicing factor EIF4A3 facilitates the biogenesis of circ-CDYL (Fig. 4A) [19], circRHBDD1 (Fig. 4B) [20], and hsa_circ_101555 (Fig. 4C) [21]. Specifically, EIF4A3 promotes the back-splicing of circRHBDD1. The resulting circRHBDD1 upregulates PIK3R1 translation via an m^6^A-dependent mechanism, activates the PI3K/AKT pathway, and elevates the expression of GLUT1 and HK2, thereby enhancing aerobic glycolysis in HCC cells. Excessive glycolysis further results in lactate accumulation and the formation of an immunosuppressive TME, which ultimately compromising the efficacy of anti-PD-1 therapy [20]. Furthermore, QKI boosts the production of circ-0008150 and circ-0007821, supporting HCC advancement [22]. Additionally, HNRNPA1 and SRSF1 promote the generation of circEPB41 (2) (Fig. 4D) and circPRKAA1 (Fig. 4E), respectively, upregulate the expression of fatty acid synthesis-related genes such as SREBP-1, ACC1, and ACLY, and accelerate fatty acid synthesis to fulfill the growth demands of HCC cells [23,24]. In contrast, the expression of QKI5 and NUDT21 is downregulated in HCC, resulting in decreased levels of tumor-suppressive circRNAs, such as circZKSCAN1 (Fig. 4F) and hsa_circ_0007874, which are regulated by these factors. This downregulation diminishes their tumor-suppressive functions [25,26].

In addition, the RNA-binding protein AUF1 facilitates circ-MALAT1 formation through back-splicing and serves dual functions. It acts as a "brake" on the ribosome, delaying the translation of PAX5 mRNA by forming a ternary complex with the ribosome and mRNA. Simultaneously, it functions as a molecular sponge, sequestering miR-6887-3p, which relieves its inhibition on JAK2 and activates the JAK2/STAT3 signaling pathway, thereby promoting the self-renewal of liver cancer CSCs (Fig. 4G) [27]. Other molecules and virus-related proteins also influence HCC progression by modulating the back-splicing of circRNAs. Remarkably, the polyadenylation complex subunit CPSF3 binds to the polyadenylation signal (PAS) sequence, inhibiting the circularization of circRNAs that contain PAS elements, such as hsa_circ_0001380. This action promotes the expression of their linear isoforms, thereby diminishing the tumor-suppressive effects mediated by miRNAs and accelerating HCC progression (Fig. 4H) [28]. Viral proteins HBx and HCV core protein modulate circ-ARL3 and circPSD3 back-splicing, contributing to virus-related HCC development [29,30].

As outlined above, diverse regulatory factors drive tumor progression by regulating the back-splicing of distinct circRNAs. These findings thus highlight promising molecular targets with great potential for the diagnosis and treatment of HCC.

#### 3.2.2 Dysregulation of AS by circRNAs

**Fig. 5:**
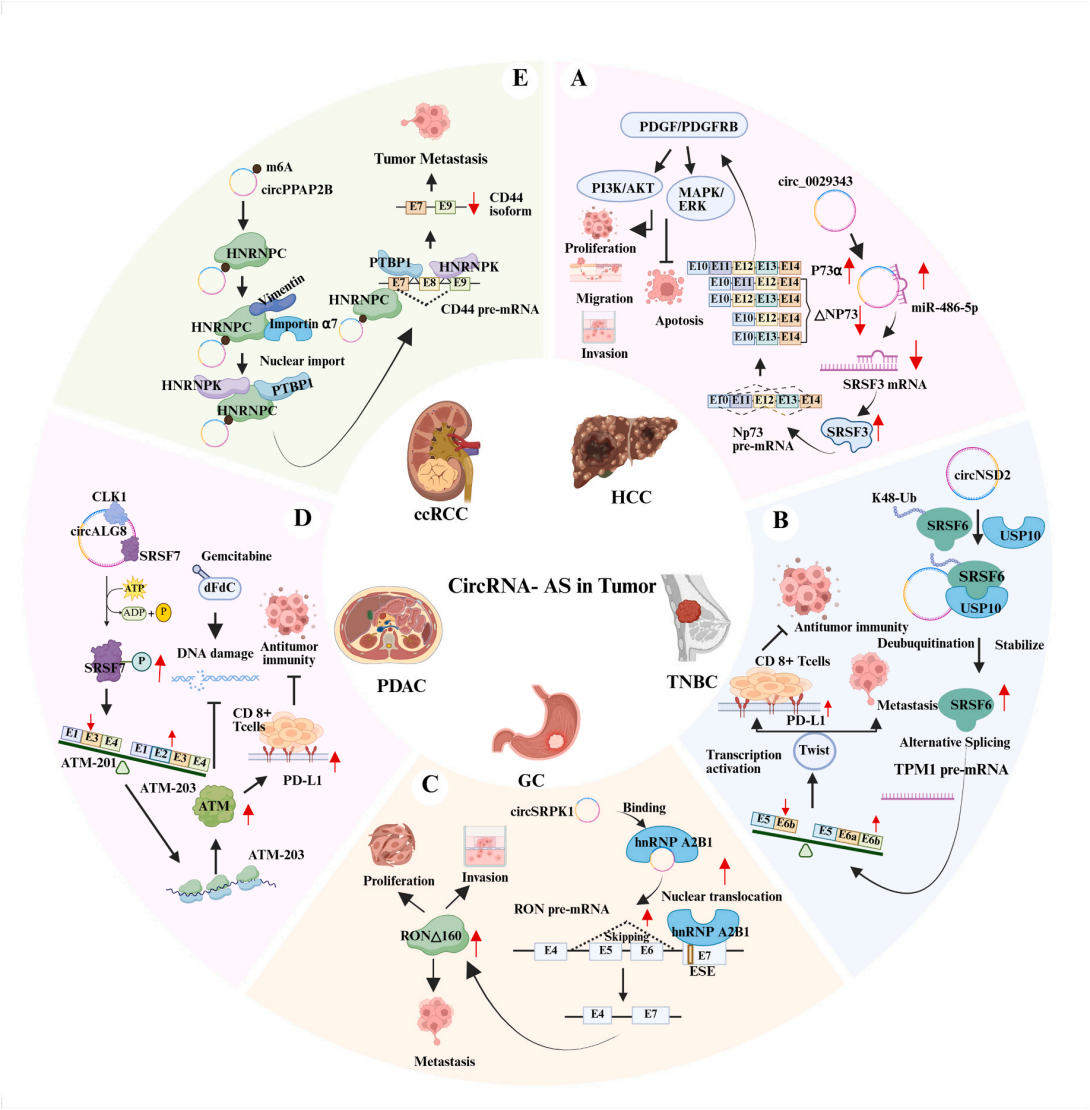
**CircRNA-mediated AS regulation in HCC and mechanistic insights from other cancers.** (A) In HCC, circRNAs act as ceRNAs to sequester miRNAs, indirectly modulating splicing factors and AS to promote tumor development. (B–E) In non-HCC malignancies—including triple-negative breast cancer (B), gastric cancer (C), pancreatic ductal adenocarcinoma (D), and clear cell renal cell carcinoma (E)—circRNAs serve as protein scaffolds that interact with splicing factors, modulating AS and tumor progression. These non-HCC examples offer valuable references for HCC studies.

Currently, studies specifically investigating circRNA-dependent AS in HCC remain relatively limited. Zhu et al. identified circ_0029343, which is significantly upregulated in HCC tissues. This circRNA acts as a miR-486-5p sponge and counteracts the inhibitory effect of miR-486-5p on splicing factor SRSF3. The elevated SRSF3 induces the splicing of the p73 gene to generate the pro-cancer subtype p73α and inhibits the production of the tumor suppressor subtype △Np73. This process thereby activates the PDGF-PDGFRB and downstream AKT/ERK signaling pathways, facilitating the growth and metastasis of HCC in vitro and in vivo (Fig. 5A) [44]. Apart from this finding, no additional mechanisms of circRNA-mediated AS regulation in HCC have been reported to date.

However, recent studies have shown that, similar to lncRNA, circRNAs function as protein scaffold by interacting with and modulating the activity of splicing factors. This mechanism has been shown to influence the development of other malignancies, such as breast cancer (Fig. 5B) [75], gastric cancer (Fig. 5C) [58], pancreatic ductal adenocarcinoma (Fig. 5D) [76], and clear cell renal cell carcinoma (Fig. 5E) [77]. As an illustration, in triple-negative breast cancer (TNBC), circNSD2 facilitates the interaction between the splicing factor SRSF6 and the deubiquitinase USP10, preventing K48-linked polyubiquitination and degradation of SRSF6. Stabilized SRSF6 promotes TPM1 exon 6a inclusion, thereby upregulating Twist to drive metastasis. Furthermore, it concurrently enhances PD-L1 expression and suppresses CD8^+^ T cell-mediated antitumor immunity, ultimately facilitating immune evasion (Fig. 5B) [75]. In gastric cancer, circSRPK1 binds to hnRNP A2B1, promoting its nuclear localization. This protein subsequently interacts with the ESE element of exon 7 in RON mRNA, inducing the skipping of exons 5 and 6 to produce the RONΔ160 isoform, which further drives tumor progression (Fig. 5C) [58]. In pancreatic ductal adenocarcinoma, circALG8 serves as a protein scaffold that facilitates the phosphorylation of SRSF7 by CLK1. This modification regulates the AS of the ATM gene, resulting in the production of the ATM203 isoform, which enhances DNA damage repair and remodels the immune microenvironment, ultimately leading to gemcitabine resistance and tumor progression (Fig. 5D) [76]. In clear cell renal cell carcinoma, circPPAP2B binds to HNRNPC in an m^6^A-dependent manner, facilitating its nuclear localization. This interaction leads to the formation of a complex with PTBP1 and HNPNPK to modulate the AS of several target genes, including CD44 (Fig. 5E) [77].

Therefore, these studies in various tumor types have established diverse mechanisms by which circRNAs modulate AS. While their direct roles in HCC splicing regulation remain to be systematically characterized, the conserved nature of these mechanisms suggests that similar regulatory axes may operate in HCC, thereby warranting dedicated investigation.

### 3.3 Crosstalk between AS and miRNAs

MiRNAs, typically 20–25 nucleotides long, play a dual role in controlling the proliferation, invasion, and drug resistance of HCC cells by targeting oncogenes or tumor suppressor genes, thus acting as pivotal regulatory elements in HCC development [78]. Recent studies have revealed that a reciprocal regulatory interplay between miRNAs and AS in HCC. AS can influence miRNA production (as summarized in Table 1). As an example, the splicing factor SLU7 enhances the formation of transcript B, housing the miR-17-92 cluster, by modulating the AS of the C13orf25 gene. This process sustains the expression of miR-17, miR-20, and miR-92a while inhibiting the expression of CDKN1A and BCL2L11, thereby promoting the survival of HCC cells [31]. Another splicing factor, hnRNPQ, collaborates with LIN28B to regulate the AS events impacting transcripts associated with the let-7 miRNA family. High expression of hnRNPQ inhibits let-7 maturation, relieves the inhibition of TRIM71, and promotes the progression of HCC [79]. Yet, the role of AS in the regulation of other miRNAs in HCC and the associated mechanisms still need further clarification.

On the other hand, through ceRNA network, miRNA exert indirect control over AS events in target genes by silencing or fine-tuning the function of splicing factors (as summarized in Table 2). For example, miR-193a-3p targets SRSF2, leading to a decrease in the pro-apoptotic form of caspase 2, thereby inducing resistance of HCC cells to 5-FU [45]. Similarly, miR-30c/e-5p suppresses SRSF10, modulating the splicing of SREK1 to favor the oncogenic isoform SREK1L, enhancing the malignant phenotype of HCC [46]. Moreover, miR-374b directly targets hnRNPA1. This interaction regulates the alternative splicing of pyruvate kinase M (PKM), thereby downregulating PKM2 expression, suppressing glycolysis, and ultimately resensitizing HCC cells to sorafenib [47]. Furthermore, miR-133b targets SF3B4 to inhibit its expression, which in turn hinders the aberrant AS of the tumor suppressor gene KLF4 mediated by the SF3B complex, thereby impeding HCC progression [48]. In addition, miR-1183 [49], miR-486-5p [50], and miR-320c [41] specifically interact with SRSF1, SRSF3, and SRSF7, respectively. These miRNAs may exert a tumor-shaping role by influencing the AS of downstream target genes; however, the specific target genes and underlying mechanisms remain to be clarified.

Taken together, the above studies underscore a reciprocal crosstalk between AS and miRNAs in HCC, which jointly modulates tumor progression and chemotherapeutic resistance. This intricate interplay deepens the molecular insights into HCC and provides new insights for targeted therapy as well.

### 3.4 Crosstalk between AS and other ncRNAs

SnoRNAs are a class of ncRNAs ranging from approximately 60 to 300 nt in length, primarily derived from the intronic regions of host genes. These molecules are categorized into two main types: the C/D box and the H/ACA box snoRNAs. Their established functions include guiding the 2′-O-methylation and pseudouridylation modifications of ribosomal RNAs (rRNAs) and small nuclear RNAs (snRNAs). Additionally, snoRNAs are involved in diverse biological processes such as rRNA acetylation, transfer RNA (tRNA) modification, regulation of mRNA stability, modulation of translation efficiency, and AS [80].

In HCC, snoRNAs play dual roles as both oncogenic and tumor-suppressive regulators. Specifically, SNORA42 [81], SNORD126 [82], SNORD52 [83], SNORA74A [84], and SNORD88B [85] facilitate the proliferation, metastasis, and self-renewal of liver cancer cells by activating signaling pathways such as PI3K/AKT, JAK2/STAT6, and Notch3, and/or by inhibiting the p53 and Hippo pathways. Conversely, SNORD13H [86] and SNORA49 [87] demonstrate tumor-suppressive effects through distinct pathways: the former by regulating rRNA modification to inhibit the RAS pathway, and the latter by interacting with HNRNPU to repress SOX9 expression.

The regulatory mechanism through which AS impacts snoRNA expression in HCC is presently unclear. In hepatoblastoma, the splicing factor PABPN1 downregulates the expression of intron-derived SNORD60 by inducing intron retention in the host gene SNHG19. This process facilitates DNA damage repair and contributes to chemotherapy resistance [88]. However, it remains to be determined whether this mechanism is also present in HCC.

SnoRNA can also participate in the progression of various tumors beyond HCC by regulating AS. As a case in point, SNORA37 modulates CD44 splicing in gastric cancer by interacting with ELAVL1 [65]. Similarly, SNORD88B influences G3BP1 splicing in trophoblast cells by recruiting the splicing factors SRSF1 and U2AF1 [89]. Additionally, SNORA70E directly binds to PARPBP pre-mRNA, inducing exon skipping to produce the pro-oncogenic subtype PARPBP-15 in ovarian cancer [90]. However, in the context of HCC, direct evidence regarding the impact of snoRNA on disease progression through the regulation of AS remains insufficient. Although previous studies have suggested that SNORA49 [87] and SNORD126 [82] in HCC can bind to the splicing factors HNRNPU and hnRNPK, respectively, their role in regulating splicing activity and AS of related genes remains to be further explored.

With a typical length of 70–90 nucleotides, tRNAs constitute a well-defined class of non-coding RNA molecules. Its primary function is to recognize mRNA codons and transport the corresponding amino acids during protein translation, thereby serving as a crucial component in the translation of genetic information [91]. Limited information is available on the reciprocal regulation between tRNAs and AS in HCC. Studies have shown that spliceosome components (SNRNP70, U2AF1, U2AF2) collaborate with Dicer1 to regulate the splicing of tRNA-Glu/TTC, resulting in the production of the tRFs fragment HCETSR. HCETSR interacts with the CH1-2 domain of SPTBN1, inhibiting its association with the β-catenin complex. This inhibition reduces the transcriptional activity of LEF1 and the expression of downstream MMP7 and CCND1, ultimately suppressing HCC proliferation and metastasis. In HCC, the low expression of tRNA-Glu/TTC itself leads to insufficient production of HCETSR, weakening its anti-cancer effect [32]. Further investigations are necessary to ascertain the potential involvement of other tRNAs in similar regulatory mechanisms.

## 4 RNA modification/editing in coupling ncRNAs and AS regulation in HCC

RNA methylation represents the most extensively studied category of RNA chemical modifications, encompassing N^6^-methyladenosine (m^6^A), 5-methylcytosine (m^5^C), N^1^-methyladenosine (m^1^A), and N^7^-methylguanosine (m^7^G), which are broadly involved in regulating RNA alternative splicing, stability, and translation efficiency [92]. Studies have demonstrated that dysregulation of RNA methylation modifications drives malignant phenotypes in HCC, including proliferation, invasion, migration, therapy resistance, and stemness maintenance [93]. Emerging findings further indicate that m^6^A modification is involved in various facets of the regulatory interplay between ncRNA-AS in HCC.

First, m^6^A modification directly modulates the AS of ncRNAs. HBx upregulates the expression of the m^6^A methyltransferase METTL3, leading to increased m^6^A levels on circ-ARL3. The m^6^A reader YTHDC1 recognizes and binds to m^6^A-modified circ-ARL3, thereby facilitating its back-splicing and biogenesis [29]. Similarly, m^6^A modification dependent on the METTL3/YTHDC1 axis accelerates the back-splicing and biogenesis of circ-CDYL. This m^6^A mark further mediates the sorting of circ-CDYL into exosomes, thereby reinforcing the stemness of HCC cells [94].

Second, m^6^A modification functions synergistically with AS-derived ncRNAs to regulate the expression of downstream target gene. CircRHBDD1, generated via alternative splicing, recruits the m^6^A reader protein YTHDF1 to PIK3R1 mRNA, thereby promoting PIK3R1 translation in an m^6^A-dependent manner [20].

Third, m^6^A modification indirectly regulates the expression of AS-related ncRNAs. In HCC, m^6^A modification mediated by METTL3, which is induced by mTORC1, enhances the stability of LINC01532. Upregulated LINC01532 binds to the splicing factor hnRNPK and promotes its phosphorylation, which in turn drives aberrant splicing of G6PD [35].

Furthermore, m^6^A modification can interplay with ncRNA-mediated AS to jointly regulate target gene expression and transcript stability. LINC01089 binds to hnRNPM, inducing the skipping of exon 3 of DIAPH3, which contains the m^6^A recognition site required for IGF2BP3 binding. IGF2BP3 binding to this site enhances *DIAPH3* mRNA stability; however, the skipping of this exon reduces m^6^A modification levels, thereby decreasing *DIAPH3* mRNA stability and subsequently downregulating its expression [34].

Overall, these findings underscore the critical role of m^6^A modification in the ncRNA-AS regulatory network and suggest that targeting RNA m^6^A modification may synergize with ncRNA-AS-directed strategies for HCC treatment. However, whether other forms of RNA modification or RNA editing contribute to the ncRNA-AS network in HCC remains largely unexplored and warrants further investigation.

## 5 Potential as diagnostic and prognostic biomarkers for HCC

### 5.1 Integrated joint analysis of tissue splicing profiles and ncRNA expression profiles

The joint analysis of multi-omics data from tissue samples is a potent approach to unveil the molecular heterogeneity of HCC and unearth new biomarkers. Through whole-genome and transcriptome sequencing of HCC tissues, driver gene mutations, copy number variations, dysregulated ncRNA expression, and abnormal AS events can be systematically identified [95]. By analyzing transcriptome sequencing data from 369 HCC and 160 normal liver samples, A total of 1375 significant alternative splicing events and 31 AS-related lncRNAs were uncovered [70]. The expression levels of these lncRNAs, including CDKN2B-AS1, were significantly correlated with patient prognosis, providing a foundation for developing prognostic models utilizing liquid biopsy or tissue analysis. However, this current study has not yet to integrate the splicing map with ncRNA expression profiles for the purpose of HCC diagnosis and prognosis assessment. The combined application of these two datasets is expected to construct a more comprehensive molecular classification system for HCC, thereby providing a critical foundation for individualized prognosis prediction and the design of precision treatment strategies.

### 5.2 Development of specific ncRNA-AS combination markers in serum/exosomes

The combination of ncRNA and AS markers shows great potential for liquid biopsy applications in HCC. NcRNAs, such as lncRNAs and circRNAs, serve as ideal sources of non-invasive biomarkers due to their stability and detectability in body fluids (e.g., serum and exosomes) [96]. Research indicates that the number of AS events of lncRNAs in serum exosomes of HCC patients is significantly lower than that in healthy controls. Notably, the splicing isoforms lncZEB2-19 and lnc-GPR89B-15 demonstrate high diagnostic efficacy in distinguishing HCC patients from healthy individuals, with area under the curve (AUC) values of 0.852 and 0.717, respectively [67]. This finding suggests that RNA splicing isoforms, which are stably present in blood, may address the sensitivity or specificity limitations of traditional markers such as alpha-fetoprotein (AFP), offering innovative non-invasive tools for early screening and differential diagnosis of HCC. Future strategies should prioritize the identification of ncRNAs associated with pathogenic splicing events, the profiling the expression of HCC-specific ncRNAs and splicing isoform alterations in serum or exosomes using high-throughput sequencing technologies, and the constructing a panel of combined markers. This strategy is expected to overcome the limitations of single markers and achieve more accurate early diagnosis and prognosis assessment of HCC.

## 6 Therapeutic strategies for targeting pathogenic splicing events

**Fig. 6:**
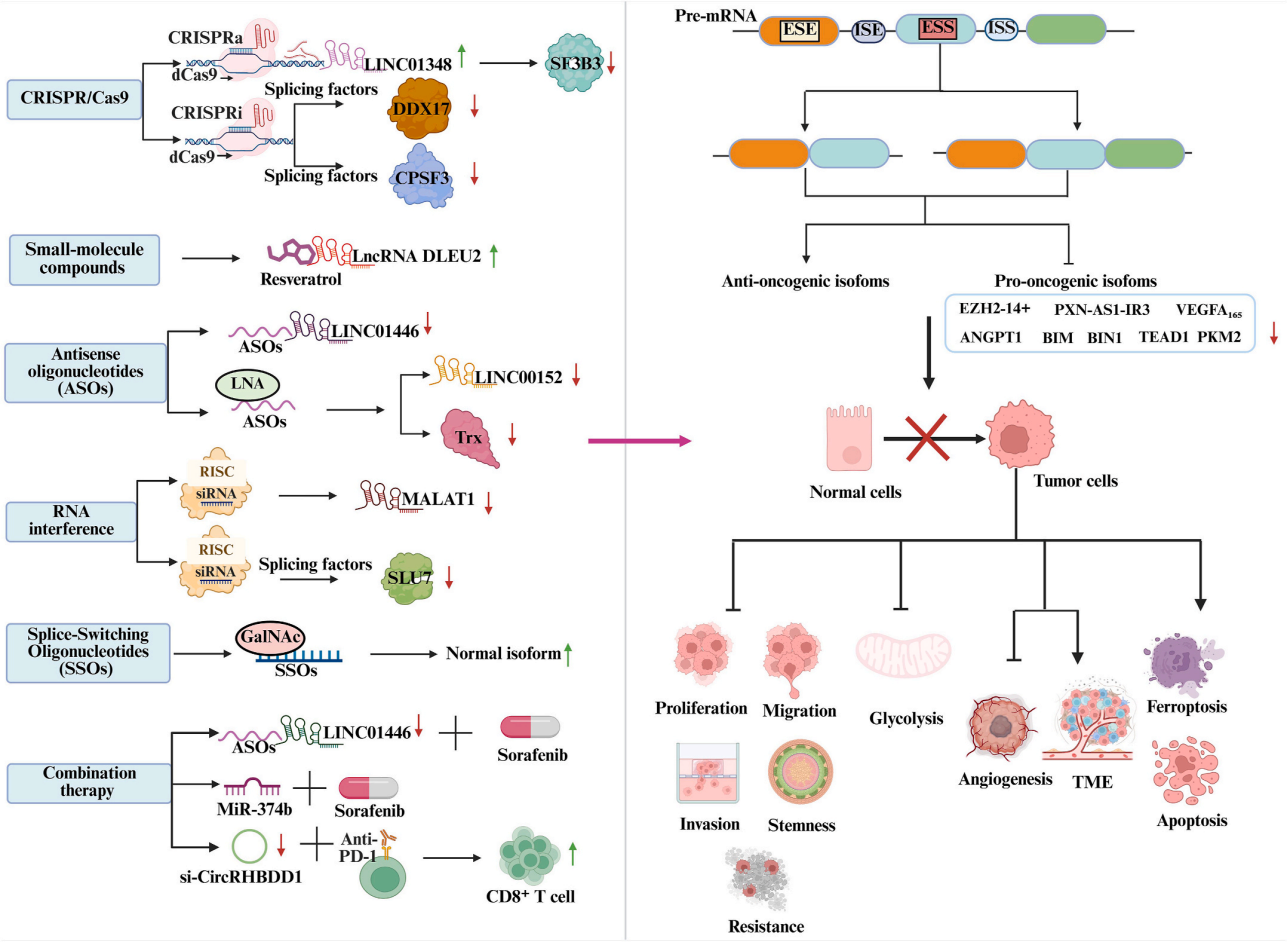
**Therapeutic targeting the ncRNA-AS axis in HCC.** Upregulation of tumor suppressors in this axis, including LINC01348, lncRNA DLEU2, and miR-339-5p, can be accomplished through CRISPRa-mediated activation or small-molecule inducers such as resveratrol. Conversely, oncogenic components such as LINC01446, LINC00152, MALAT1, SLU7, DDX17, and Trx can be suppressed using CRISPRi, RNA interference, or antisense oligonucleotides (ASOs). Splicing-switching oligonucleotides (SSOs) may correct aberrant alternative splicing events in HCC. Moreover, ncRNA-AS axis-targeted therapies may be administered in combined with sorafenib and anti-PD-1 immunotherapy. All the above approaches downregulate the expression of oncogenic splice isoforms (EZH2-14+, PXN-AS1-IR3, VEGFA_165_, ANGPT1, BIM, BIN, TEAD1 and PKM2), thereby suppressing HCC cell proliferation, stemness, invasion, migration, angiogenesis and glycolysis, remodeling the tumor immune microenvironment, and triggering apoptosis and ferroptosis in HCC cells.

Accumulating studies suggest that multiple strategies targeting the ncRNA-AS axis hold therapeutic promise for HCC (as summarized in Fig. 6). These include CRISPRa-mediated activation or small-molecule induction of tumor suppressors within key hub genes, as well as CRISPRi, RNA interference, antisense oligonucleotides (ASOs), and pharmacological inhibition of oncogenic components in the same network. Additionally, the correction of aberrant alternative splicing via splice-switching oligonucleotides (SSOs) represents another viable approach. Furthermore, combining ncRNA-AS axis-targeted therapies with established treatments such as sorafenib or anti-PD-1 immunotherapy may synergistically enhance antitumor efficacy. Thus, these findings underscore the potential of multifaceted interventions targeting the ncRNA-AS axis for HCC management.

### 6.1 Targeting critical regulatory hubs of ncRNA and splicing factors

Targeting key ncRNAs and splicing factors within the ncRNA–AS regulatory network has emerged as an innovative strategy for HCC therapy. Currently, the major approaches to upregulate tumor-suppressive ncRNAs involved in splicing regulation include CRISPR/Cas9 gene editing and small-molecule compounds [36,97,98]. Of note, activation of LINC01348 via the CRISPRa system, which fuses catalytically dead Cas9 (dCas9) with the transcriptional activator VP64, suppresses the migration of HCC cells in vitro, although its anti-tumor efficacy in vivo remains to be further validated [36]. Small-molecule compounds also exhibit considerable regulatory potentia. Resveratrol upregulates multiple splice variants of the tumor-suppressive lncRNA DLEU2 in mouse hepatocytes [97], yet its functional significance in HCC remains unclear. Kaempferol increases miR-339-5p expression in colon cancer, inhibits the splicing factors hnRNPA1 and PTBP1, reduces PKM2 production, and thereby suppresses tumor cell proliferation [98]. Nonetheless, its functional roles in HCC require further investigation.

In contrast, more strategies have been developed to downregulate oncogenic ncRNAs and splicing regulators, mainly including antisense oligonucleotides (ASOs), RNA interference (e.g., siRNA), and CRISPR/Cas9 gene editing. Among these, ASO intervention has demonstrated notable therapeutic potential in HCC models [38,42]. ASOs targeting LINC01446 inhibit the production of the oncogenic VEGFA_165_ splice variant and effectively suppress HCC growth in vitro and in vivo [38]. Teng X et al. further applied locked nucleic acid (LNA)-modified ASOs with enhanced stability and efficacy to target LINC00152 and the RBPs Trx, reducing the generation of oncogenic ANGPT1 isoforms and inhibiting HCC cell invasion and migration [42]. RNA interference-mediated knockdown of MALAT1 reduces the expression of oncogenic splice isoforms of BIM, BIN1, and TEAD1, thereby suppressing HCC cell proliferation [39]. By comparison, studies employing CRISPR/Cas9 editing to modulate AS-related ncRNAs for HCC therapy remain scarce, warranting in-depth mechanistic investigations.

Regarding the targeting of splicing factors, siRNA-mediated knockdown of SLU7 promotes autophagy-dependent apoptosis in HCC cells accompanied by reactive oxygen species accumulation, thus restraining HCC progression [31]. CRISPR/Cas9-mediated knockout of the splicing factors DDX17 and CPSF3 also exerts potent anti-tumor effects: ablation of DDX17 suppresses the production of the oncogenic PXN-AS1-IR3 splice isoform [16], while CPSF3 depletion modulates the back-splicing of hsa_circ_0001380 and hsa_circ_0078607, both of which inhibit HCC cell proliferation and migration [28]. JTE-607, a CPSF3 inhibitor, also suppresses HCC cell growth in vitro and in vivo by regulating CPSF3-dependent circRNAs [28]. Notably, small molecules directly targeting the core splicing machinery—including pladienolide B (a spliceosome inhibitor) and CCT018159 (a splicing factor inhibitor)—also exhibit efficacy in suppressing HCC cell proliferation and tumor growth in vivo [99,100]. However, whether these direct splicing modulators exert anti-HCC effects by regulating AS events of ncRNAs remains to be fully elucidated, providing a promising direction for the development of multi-target synergistic therapeutic strategies.

Collectively, intervention strategies targeting key nodes of the ncRNA–AS regulatory network have shown promising potential for HCC treatment. Despite this, the clinical translation of these approaches, ranging from small molecules to gene-editing technologies, still requires further mechanistic dissection and translational research.

### 6.2 Therapeutic correction of pathogenic splicing defects

Directly correcting pathogenic splicing events caused by dysregulation of ncRNAs or splicing factors is a precise therapeutic strategy. Splice-switching oligonucleotides (SSOs) are a type of synthetic antisense oligonucleotides that can bind to splicing sites or regulatory elements on pre-mRNA via complementary base pairing, blocking the binding of splicing factors. This interaction induces exon skipping, intron retention, or the exclusion of pseudo-exons, ultimately leading to the production of functionally normal protein isoforms or facilitating the degradation of pathogenic isoforms. SSOs represent innovative therapeutic interventions for addressing RNA splicing disorders [101]. In solid tumors, the SSO strategy has shown therapeutic potential. In pancreatic ductal carcinoma, SSOs targeting the 5′ untranslated region (5′UTR) of the AURKA gene inhibit SRSF1-mediated inclusion of Alu exons, thereby disrupting the oncogenic loop involving SRSF1, AURKA, and MYC. These SSOs also decrease cell viability and induce apoptosis [102]. In prostate cancer, SSOs facilitate exon 4 skipping of the ERG gene, leading to a significant downregulation of the ERG protein and a subsequent inhibition of cell proliferation and migration [103]. Although direct research on SSOs targeting HCC is still limited, advancements in relevant delivery technologies offer a promising translational foundation. The cross-linked low-molecular-weight linear polyethylenimine nanocarrier can efficiently deliver SSOs to the liver cell nucleus, enabling liver-specific splicing correction [104]. Moreover, GalNAc-modified SSOs can restore the expression of normal splicing isoforms of SLC25A13 in hepatocytes [105], indicating the feasibility of this approach for HCC cells. As HCC-specific ncRNA splicing targets are progressively unveiled, SSOs are expected to promote the development of precise treatment for HCC.

### 6.3 Combined targeting of ncRNA-AS and other anti-HCC compounds

Combination therapy targeting ncRNA-AS alongside traditional small molecule compounds or immunosuppressants is increasingly recognized as a pivotal strategy to break through bottlenecks in cancer treatment. This approach paves the way for more precise therapies for malignant tumors, including HCC. Studies demonstrate that co-administration of LINC01446-targeting ASO and sorafenib significantly reduces tumor volume and microvascular density in HCC xenograft models. This effect is achieved by concurrently inhibiting angiogenesis through two distinct mechanisms: the ASO degrades lncRNA, which blocks the AS of VEGFA_165_, while sorafenib inhibits the RAF/MEK/ERK signaling pathway [38]. By co-targeting angiogenic splice variants and kinase cascades, the two agents synergistically inhibit HCC progression across two distinct molecular axes, laying a robust mechanistic foundation for combination therapy. MiR-374b increases the sensitivity of HCC cells to sorafenib treatment by regulating the AS of PKM2. When administered alongside sorafenib, it produces a synergistic therapeutic effect against HCC, both in vivo and in vitro [47]. In addition, the knockdown of circRHBDD1, the back-splicing product mediated by EIF4A3, also inhibits aberrant glycolysis, which markedly enhances CD8^+^ T cell infiltration within cancerous tissues, consequently elevating the efficacy of immunotherapy [20]. Both ncRNA-AS regulators converge to correct metabolic rewiring to overcome treatment insensitivity, which complements the anti-proliferative, anti-angiogenic or immune-stimulating effects of traditional therapies. This complementary regulatory mechanism generates synergistic anti-tumor activity without merely additive toxicity. This finding provides crucial empirical support for the integration of ncRNA-AS targeted therapy with immunotherapy. As delivery mechanisms are optimized and clinical investigations progress, this therapeutic approach is poised to become a conventional intervention for diverse malignancies, ultimately leading to improved patient outcomes.

### 6.4 Unanswered questions in clinical translation

Clinical translation in the ncRNA-AS regulatory network in HCC therapy is still confronted with several key challenges. First, the understanding of the ncRNA-AS regulatory network in HCC remains incomplete. At present, studies on the role of AS in lncRNAs, miRNAs, snoRNAs, and tRNAs in HCC are relatively scarce. Meanwhile, research on the molecular mechanisms and temporal patterns of AS regulation in HCC by ncRNAs other than lncRNAs is also insufficient, and their dynamic regulatory modes under different tumor microenvironments (such as hypoxia and inflammation) still need in-depth exploration [106]. Second, HCC displays considerable molecular heterogeneit. There are obvious differences in ncRNA expression profiles and AS patterns among HCC patients with different etiologies and subtypes, which greatly restricts the universality and individualized adaptability of strategies targeting the ncRNA-AS regulatory network [107,108]. Third, the regulatory network is intricate and functionally redundant, providing substantial robustness to the network through mechanisms such as homologous genes and shadow enhancers. Single-target intervention often has limited effects and is easily offset by the activation of compensatory signaling pathways [[109], [110], [111]]. Fourth, the in vivo delivery efficiency and targeting specificity of oligonucleotide drugs (such as ASOs and SSOs) still need to be improved. These molecules are easily degraded by nucleases and difficult to effectively break through the hepatic first-pass effect and tumor tissue penetration barrier. Although delivery strategies such as nanocarriers have shown application potential, they still need further optimization [112,113]. Fifth, there is a high risk of potential off-target effects. Splicing factors exert extensive regulatory control, and targeted intervention may disrupt the transcriptome, leading to off-target toxicities such as liver injury and myelosuppression. For instance, although SF3B1 inhibitors can effectively inhibit HCC cell proliferation, they may also affect the function of normal hepatocytes and pose a risk of inducing liver injury [114]. Sixth, the evidence supporting clinical translation is insufficient. Most targeted strategies remain at the preclinical stage and lack validation through large-scale, multi-center clinical studies [110]. Meanwhile, existing animal models are difficult to accurately simulate the real human tumor microenvironment and splicing regulatory network, resulting in in some intervention effects that cannot be reproduced [115].

## 7 Conclusion and perspectives

In summary, ncRNAs and AS form a highly dynamic and deeply intertwined bidirectional regulatory network in HCC. This network, far from being a straightforward causal chain, significantly propels the malignant progression of HCC through intricate interactions. Dysregulated ncRNAs act as upstream regulators, reshaping the splicing landscape of HCC cells by influencing splicing factors, kinases, and RBPs. This process leads to the production of oncogenic and tumor-suppressive protein isoforms. Simultaneously, abnormal AS events in HCC can also regulate the generation and function of ncRNA isoforms, which are pivotal in essential biological processes such as cell proliferation, stemness maintenance, invasion and metastasis, metabolic reprogramming, drug resistance, and tumor microenvironment remodeling. Various studies complement each other, gradually improving the comprehensive understanding of this regulatory network, shedding light on its multifaceted functions.

In the future, a systems biology approach that integrates single-cell multi-omics and computational modeling is essential to unravel the specific core regulatory circuits of HCC and identify therapeutic targets with functional significance and intervention feasibility. Emphasis should be placed on innovative delivery systems, such as nanocarriers and exosomes, to improve the liver specificity and tissue penetration efficiency of targeted drugs, addressing the in vivo delivery challenges of oligonucleotide drugs. Utilizing high-throughput sequencing and artificial intelligence, a personalized molecular typing model should be developed, incorporating ncRNA expression profiles and splicing maps to tailor precise treatment strategies. Concurrently, gene editing and splicing intervention technologies such as CRISPR/Cas9 and SSO, should be employed to target dysregulated ncRNAs and pathogenic splicing isoforms, facilitating the translation of fundamental research findings into novel strategies for the precise diagnosis and treatment of HCC. The full names of partial abbreviations are presented in Table 3.

**Table 3:** Glossary of abbreviations.

Abbreviation	Full form	Abbreviation	Full form
ACC1	Acetyl-CoA Carboxylase 1	ISS	Intronic Splicing Silencer
ACLY	ATP-Citrate Lyase	KLF4	Kruppel Like Factor 4
AMPK	AMP-Activated Protein Kinase	LNA	Locked Nucleic Acid
ANGPT1	Angiopoietin 1	LEF1	Lymphoid Enhancer Binding Factor 1
ATM	Ataxia Telangiectasia Mutated	LIN28 A/B	Lin-28 Homolog A/B
AURKA	Aurora Kinase A	MBNL3	Muscleblind-Like-3
BIM	BCL2 Interacting Mediator of Cell Death	MEIS2	Meis Homeobox 2
BIN1	Bridging Integrator 1	MMP2/7	Matrix Metallopeptidase 2/7
CCAR1	Cell Cycle And Apoptosis Regulator 1	NCAPG2	Non-SMC Condensin II Complex Subunit G2
CCND1	Cyclin D1	NONO	Non-POU Domain Containing Octamer Binding Protein
CDCA3/7	Cell Division Cycle Associated 3/7	NUDT21	Nudix Hydrolase 21
CDKN1A	Cyclin Dependent Kinase Inhibitor 1A	PABPN1	Polyadenylate-binding Protein Nuclear 1
COL4A1	Collagen Type IV Alpha 1 Chain	PAX5	Paired Box 5
CPSF3	Cleavage and Polyadenylation Specific Factor 3	PAS	Polyadenylation Signal
CREB1	cAMP Responsive Element Binding Protein 1	PRKAA1	Protein Kinase AMP-Activated Catalytic Subunit Alpha 1
Ct-HBx	C-Terminal Truncated Hepatitis B Virus X Protein	PTBP1	Polypyrimidine Tract Binding Protein 1
DDX17	DEAD-Box Helicase 17	PXN	Paxillin
DEAS	Differentially expressed alternative splicing events	QKI5	Quaking RNA Binding Protein Isoform 5
EEF1A2	Eukaryotic Translation Elongation Factor 1 Alpha 2	RON	Recepteur d’Origine Nantais
EIF4A3	Eukaryotic Initiation Factor 4A3	RRM	RNA Recognition Motif
ESE	Exonic Splicing Enhancer	RBM5	RNA Binding Motif Protein 5
ESS	Exonic Splicing Silencer	S6K1	Ribosomal Protein S6 Kinase 1
EVA1A	Eva-1 Homolog A	SCD1	Stearoyl-CoA Desaturase 1
ESRP2	Epithelial Splicing Regulatory Protein 2	SF3B3	Splicing Factor 3B Subunit 3
FAS	Fas Cell Surface Death Receptor	SNHG19	Small Nucleolar RNA Host Gene 19
FASN	Fatty Acid Synthase	SLC22A3	Solute Carrier Family 22 Member 3
FMRP	Fragile X Mental Retardation Protein	SLC25A13	Solute Carrier Family 25 Member 13
FSP1	Ferroptosis suppressor Protein 1	SSOs	Splice Switching Oligonucleotides
FTO	Fat Mass and Obesity-Associated Protein	SR	Serine/Arginine-Rich Splicing Factor
G6PD	Gglucose-6-phosphate dehydrogenase	SRPK1/2	Serine-arginine Protein Kinase 1/2
GLUT	Glucose Transporter	SREBP-1	Sterol Regulatory Element Binding Protein 1
H3K27ac	Histone H3 Lysine 27 Acetylation	SREK1	Splicing Regulatory Glutamic Acid/Lysine-Rich Protein 1
H3K4me3	Histone H3 Lysine 4 Trimethylation	SRSF	Serine/Arginine-Rich Splicing Factor 1
HDLBP	HDL Binding Protein	TPM1	Tropomyosin 1
HK2	Hexokinase 2	TRIM69/71	Tripartite Motif Containing Protein 69/71
hnRNP	Heterogeneous Nuclear Ribonucleoprotein	UBXN7	UBX Domain Protein 7
ISE	Intronic Splicing Enhancer	USP10/21	Ubiquitin Specific Peptidase 10/21

## Funding

This work was supported in part by grants from 10.13039/100012547Natural Science Foundation of Guangxi Zhuang Autonomous Region [Grant Number: 2025GXNSFHA069034] and from High-level Talent Program of Youjiang Medical University for Nationalities [Grant Number: yy2019rcky008].

## CRediT authorship contribution statement

**Delong Wang:** Data curation, Visualization, Writing – original draft. **Jingli Zhong:** Conceptualization, Visualization, Writing – review & editing. **Zhangxiu Liao:** Conceptualization, Funding acquisition, Writing – original draft, Writing – review & editing.

## Declaration of competing interest

The authors declare that they have no known competing financial interests or personal relationships that could have appeared to influence the work reported in this paper.
